# The role of the amygdala in depression: a bibliometric analysis (2015–2024)

**DOI:** 10.3389/fpsyt.2025.1642936

**Published:** 2025-11-13

**Authors:** Yuanyuan Li, Dengxian Yang, Mengye Cao, Lifang Dong, Liuyin Jin, Shugui Gao

**Affiliations:** 1 School of Medicine, Ningbo University, Ningbo, Zhejiang, China; 2 The Affiliated Kangning Hospital of Ningbo University, Ningbo, Zhejiang, China; 3 Nursing Department, Ningbo College of Health Sciences, Ningbo, Zhejiang, China; 4 Lishui Second People’s Hospital, Lishui, Zhejiang, China

**Keywords:** depression, amygdala, bibliometrics, emotion regulation, resting-state functional connectivity, neural mechanisms

## Abstract

**Background:**

Depression is a highly heterogeneous disorder with complex mechanisms. Given converging evidence implicating the amygdala in its pathophysiology, a systematic and quantitative synthesis is warranted to map the research landscape, hotspots, and emerging trends.

**Objective:**

To systematically characterize the research landscape of depression–amygdala studies from 2015 to 2024 using bibliometric and visualization analyses, identify core hotspots and emerging themes, and track their evolution to inform subsequent mechanistic research and precision interventions.

**Methods:**

Using data from the Web of Science Core Collection and Scopus (2015–2024), we conducted a bibliometric analysis of English-language publications on depression and the amygdala with *Bibliometrix* and *VOSviewer*. Publication trends, country and institutional contributions, highly cited papers, and keyword co-occurrence/clustering were analyzed to delineate the field’s structure and evolution.

**Results:**

A total of 5,999 publications were included. Annual output increased steadily from 399 in 2015 to a peak of 831 in 2024. The United States (1,813, 30.2%), China (1,122, 18.7%), and Germany (357, 6.0%) were the top contributors. The ratio of multi-country publications (MCP) was highest for Germany (28.3%), followed by the United Kingdom (28.1%) and Canada (24.6%). At the institutional and journal levels, the University of California system, Harvard University, and Harvard Medical School ranked among the leading contributors. The international collaboration network exhibited a U.S.-centered structure, with frequent partnerships between the United States and China (n = 113), the United States and the United Kingdom (n = 86), and the United States and Germany (n = 81). Keyword co-occurrence and clustering analyses revealed four major thematic clusters: emotion regulation networks, biological signaling and regulation, developmental risk factors, and animal models. Overall, the field has evolved from region-specific studies toward network- and system-level integration, highlighting the amygdala’s pivotal role in the neurobiological mechanisms of depression and its potential as a candidate neuromarker.

**Conclusion:**

Bibliometric evidence indicates that research on depression–amygdala relationships is moving toward multi-dimensional and cross-disciplinary integration. The amygdala’s involvement in emotion regulation and early-life stress is increasingly recognized; alterations in its functional connectivity show promise as a neuromarker of depression, though clinical translation requires multi-center validation and standardized analytic workflows.

## 1 Introduction

Mental disorders are a group of conditions characterized by persistent disturbances in emotion, cognition, or behavior that significantly impair functioning in learning, work, and interpersonal life. Major categories include depressive disorders, bipolar and related disorders, anxiety disorders, trauma- and stressor-related disorders, obsessive–compulsive and related disorders, psychotic disorders, and substance use disorders (1). Among these, depressive disorders are the most prevalent and represent major causes of disability and socioeconomic burden, thereby attracting sustained attention from both society and the scientific community (1–3). Depression is a common psychiatric disorder characterized by persistent low mood, loss of interest or pleasure, and reduced motivation, often accompanied by cognitive impairment, sleep disturbances, appetite changes, and other physiological or psychological symptoms (4). It severely affects emotional well-being, daily functioning, and overall quality of life, imposing substantial burdens on social and occupational performance (5). In recent years, the global prevalence of depression has continued to rise, with high relapse and disability rates making it one of the most pressing public health challenges worldwide (6). According to the World Health Organization (WHO), over 350 million people are affected by depression globally, with notably high incidence among adolescents, the elderly, and women (7). Forecasts suggest that by 2030, depression will rank among the top three causes of global disease burden, particularly in low- and middle-income countries where its societal and health impacts are more pronounced (8). Despite ongoing advances in research, clinical diagnosis still primarily relies on interviews and self-report scales, with a lack of objective and quantifiable biological markers (9). Consequently, identifying brain regions with potential as biomarkers—particularly those exhibiting functional abnormalities—has become a critical direction in elucidating the pathophysiology of depression.

Among these regions, the amygdala has garnered significant attention due to its pivotal role in emotion and cognitive processing. As a core structure of the limbic system, the amygdala is extensively connected to the cerebral cortex, subcortical areas, and brainstem, functioning as a hub in brain networks that support emotion regulation, learning, memory consolidation, attention allocation, and executive function (10, 11). In emotional processing, the amygdala is highly responsive to negative emotions such as fear and anger, rapidly activating upon exposure to threatening stimuli and initiating autonomic responses that modulate affective states (10, 12). In memory-related functions, the amygdala plays a key role in encoding and consolidating emotionally salient memories, particularly by interacting with the hippocampus and modulating neurotransmitter activity (13). While these functions support environmental adaptation, they may become dysregulated in pathological conditions. Neuroimaging studies indicate that, in response to negative emotional stimuli, patients with depression commonly exhibit amygdala hyperactivation (14–22), accompanied by reduced functional connectivity between the amygdala and prefrontal cortical regions (23–25). This neural phenotype is consistent with the negative information-processing bias, deficits in emotion regulation, and alterations in emotional memory observed in depression, and may contribute to the persistence and exacerbation of symptoms (26–29). As such, amygdala dysfunction is considered a central node in the neural mechanisms of depression and a potential target for intervention and neuroimaging-based biomarker development (30).

In recent years, bibliometric analysis—an approach based on publication, citation, and text-mining data—has been increasingly employed to assess research trends and scientific landscapes across disciplines (31). By quantifying relationships among authors, institutions, keywords, and publications, bibliometric methods reveal developmental trajectories, research hotspots, and the knowledge structure of a given field (31). This includes not only descriptive statistics such as publication volume and citation frequency but also advanced network analyses such as keyword co-occurrence, collaboration networks, and document clustering (32). Based on this, the present study focuses on literature addressing “amygdala” and “depression” from 2015 to 2024, employing bibliometric and visualization approaches using *VOSviewer*, *ggplot2*, and *Bibliometrix*. Through analyses of publication trends, country and institutional contributions, high-frequency keywords, co-occurrence networks, and literature clustering, this study aims to systematically map the central role of the amygdala in depression research, identify thematic hotspots, and elucidate the field’s evolutionary patterns. The findings are expected to provide theoretical and data-driven support for future mechanistic studies and the development of optimized intervention strategies. Meanwhile, advances in artificial medical intelligence and the Internet of Medical Things (IoMT) have opened avenues for multimodal integration of genetic and hormonal indicators with neural circuit–level readouts (33, 34). This technological convergence complements the amygdala-centered perspective of the present study and provides a foundation for hypothesis generation and translational exploration.

This study makes four key contributions. First, it merges and deduplicates Web of Science and Scopus records from 2015 to 2024 and applies a reproducible bibliometric workflow to map amygdala-related depression research. Second, it quantifies the global structure by characterizing a collaboration network centered on the United States, and by examining differences in the proportion of multi-country publications, thereby linking research output to patterns of knowledge flow. Third, it robustly identifies four major themes—emotion regulation networks, molecular and neural regulation, developmental risk factors, and animal models—demonstrating a shift toward system-level integration. Fourth, it highlights amygdala functional connectivity as a potential neuromarker for patient stratification and treatment prediction.

## 2 Methods

### 2.1 Data sources and literature search strategy

This bibliometric analysis was conducted using the Web of Science Core Collection (*WoS*) and *Scopus* databases—two of the most authoritative and widely used academic literature repositories (35). WoS indexes approximately 12,000 high-impact journals and is known for its precise citation tracking, standardized data, and broad subject coverage (36). Scopus includes over 24,000 journals and is recognized for its strong subject representation and robust search capabilities (37). Both databases adopt rigorous journal selection criteria, ensuring high data quality, credibility, and reproducibility. To enhance the comprehensiveness and robustness of the results, both WoS and Scopus were used as data sources in this study.

**Figure 1:**
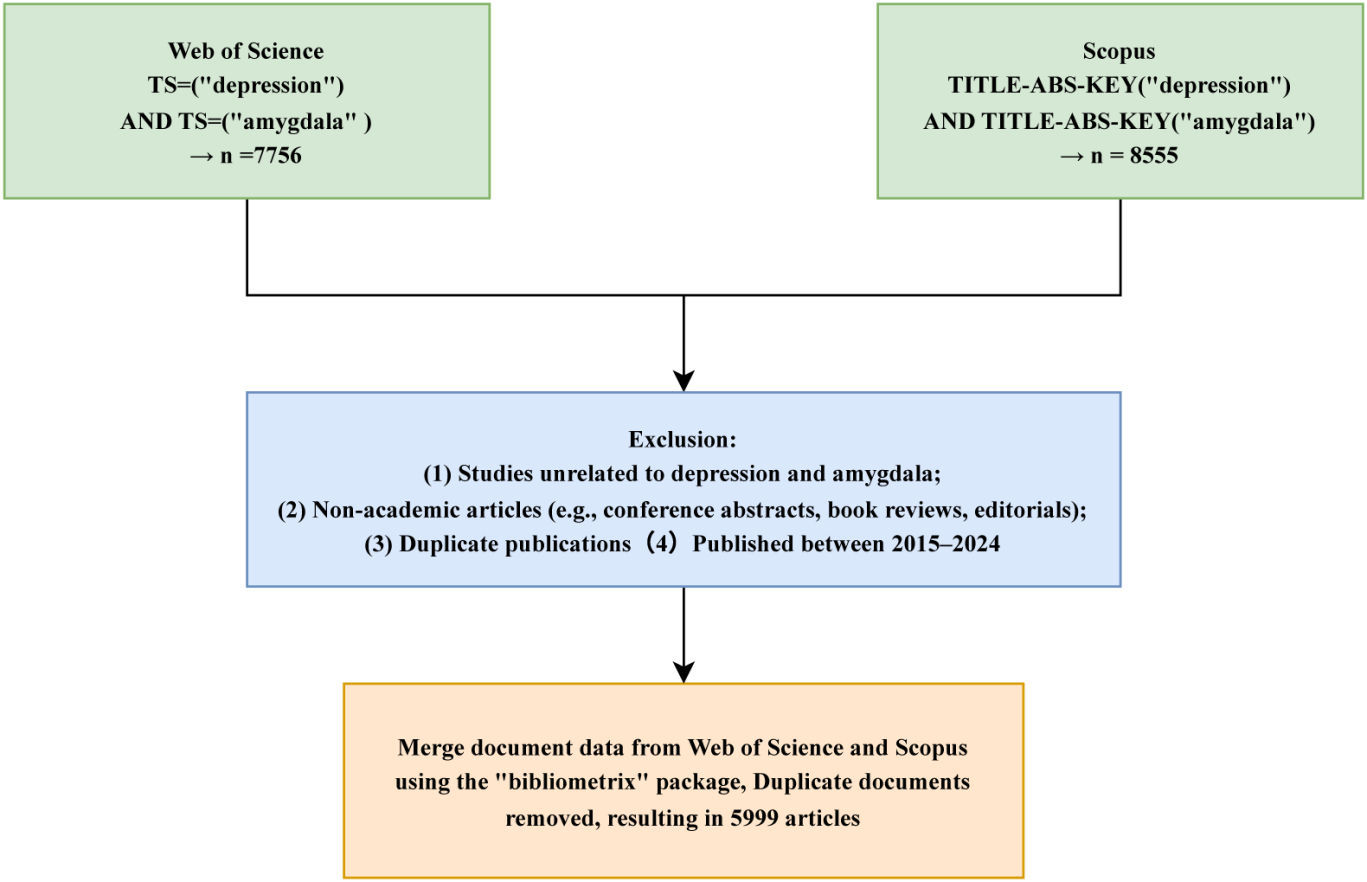
Flowchart of literature screening and data processing for bibliometric analysis.

The search strategy focused on the keywords “depression” and “amygdala,” using the query *TS = (“depression”) AND TS = (“amygdala”)* for WoS and *TITLE-ABS-KEY = (“depression”) AND TITLE-ABS-KEY = (“amygdala”)* for Scopus. The inclusion criteria were as follows (1): English-language publications (2); document type limited to “Article”; and (3) publication years between 2015 and 2024. Exclusion criteria included conference abstracts, proceedings, book reviews, editorials, and other non-peer-reviewed materials. All searches and data exports were completed on April 27, 2025, to ensure consistency and reproducibility. Exported records were saved in*.txt* format, including core metadata fields such as article titles, authors, publication years, journal names, keywords, abstracts, and citation counts. The literature selection process is illustrated in **Figure 1**. The time window was restricted to 2015–2024 to enhance comparability within the DSM-5 diagnostic framework and to reflect the relative stability of MRI acquisition and preprocessing pipelines, while focusing on contemporary research trends.

### 2.2 Literature screening and data processing

After the initial data export, all records were manually reviewed and cleaned. Publications not meeting the inclusion criteria were removed, and duplicates were eliminated. Only peer-reviewed original research articles and review papers were retained. Records from the two databases were subsequently merged, and duplicate entries were systematically removed. The final dataset included information pertaining to key bibliometric indicators, such as publication volume, total citations, keyword distribution, author and collaboration networks, research institutions, countries/regions, journal sources, and cited references. In addition, cross-disciplinary studies have demonstrated the effectiveness of systems mapping combined with network visualization in identifying thematic structures and tracing knowledge evolution (38). Bibliometric research in the Internet of Medical Things (IoMT) domain has likewise demonstrated the feasibility of network-based hotspot detection and topic evolution tracking (39), providing methodological precedent and external validation for the visualization framework used in this study.

### 2.3 Data analysis and visualization

To comprehensively identify developmental trajectories, research hotspots, and emerging themes in the field of depression and the amygdala, this study employed a combination of bibliometric tools and visualization software, including *Bibliometrix* (v4.4.3), *ggplot2* (v3.5.1), and *VOSviewer* (v1.6.19). *Bibliometrix* (v4.4.3) was used to perform fundamental bibliometric analyses, including annual publication trends, author and institutional productivity, citation frequencies, journal distributions, and country-level contributions. It also generated time-series plots, collaboration networks, and thematic evolution maps (31). *ggplot2* (v3.5.1) was utilized to enhance the visual presentation of results, generating trend charts, heatmaps, and keyword distribution plots for improved clarity and aesthetic quality (40). *VOSviewer* (v1.6.19) was employed to construct keyword co-occurrence networks, co-citation networks, and cluster visualizations (41). In these network maps, node size represents keyword frequency or the number of publications, edge thickness indicates co-occurrence or co-citation strength, and node color differentiates between clusters or time periods.

## 3 Results

### 3.1 Annual publication trends

**Figure 2:**
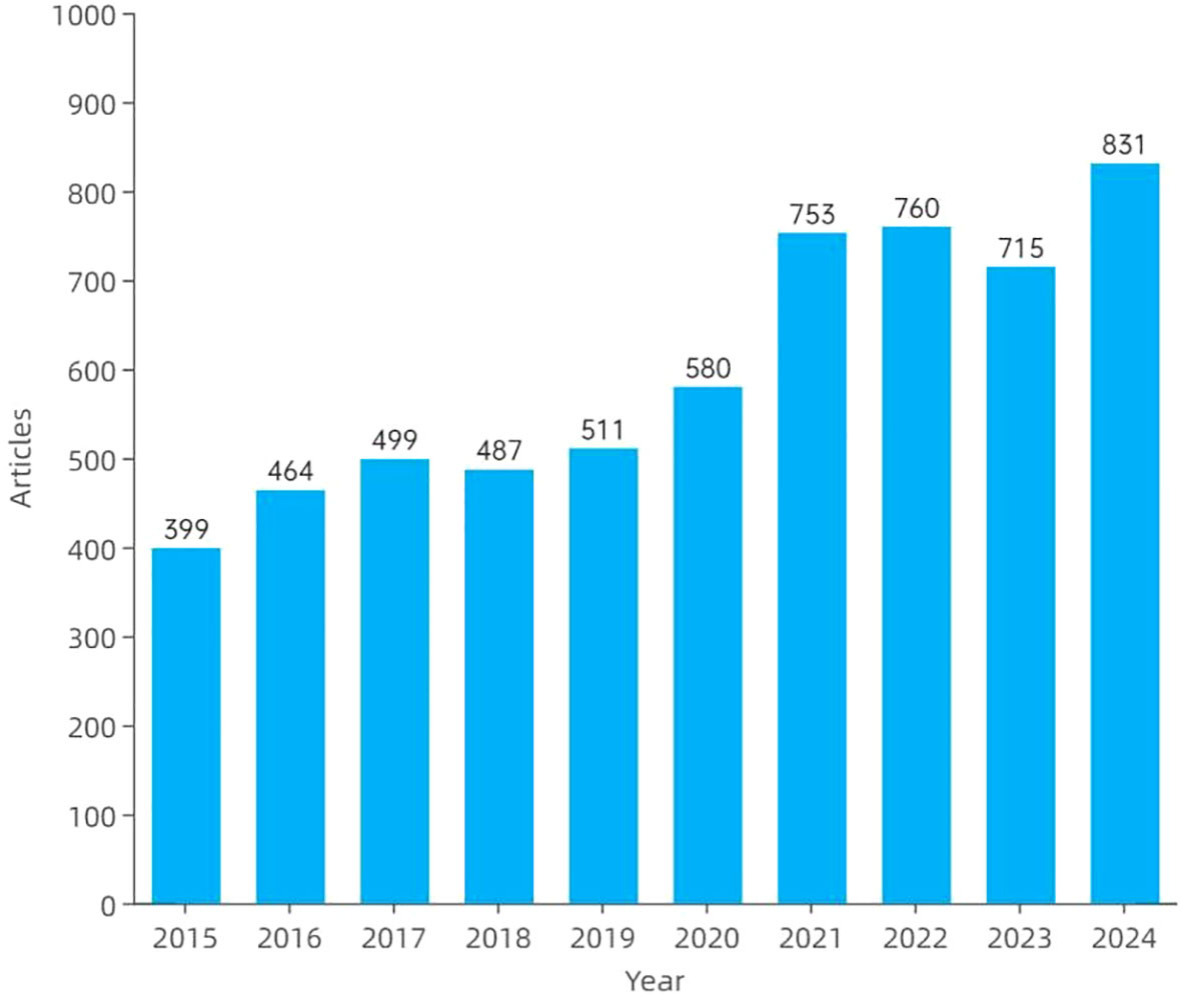
Annual publication count on amygdala and depression research from 2015 to 2024.

**Figure 2** illustrates the annual publication trends in amygdala–depression research from 2015 to 2024. A total of 5,999 publications were included, consisting of 4,819 original articles and 1,180 review papers. Overall, the number of publications showed a consistent upward trajectory, increasing from 399 articles in 2015 to 831 in 2024. The growth was particularly evident from 2020 onwards, with 753 and 760 articles published in 2021 and 2022, respectively. Although a slight decline was observed in 2023, the number of publications rebounded in 2024, reaching an all-time high.

### 3.2 Analysis of country, institution, journal, and author distributions

In the field of amygdala–depression research, scientific output showed a marked concentration across countries, institutions, journals, and authors. Among 71 countries and regions, the United States ranked first in publication output (N = 1,813, 30.2%), followed by China (N = 1,122, 18.7%) and Germany (N = 357, 6.0%). Germany showed the highest rate of multiple-country publications (MCP = 28.3%), indicating stronger engagement in international collaboration, followed by the United Kingdom (28.1%) and Canada (24.6%) (**Table 1**).

**Table 1:** Top 10 countries or regions by depression and amygdala publication volume from 2015 to 2024.

Country	Articles	Articles %	SCP	MCP	MCP%
USA	1813	30.2	1620	193	10.6
China	1122	18.7	984	138	12.3
Germany	357	6	256	101	28.3
Canada	240	4	181	59	24.6
United Kingdom	221	3.7	159	62	28.1
Italy	189	3.2	157	32	16.9
Japan	184	3.1	168	16	8.7
Australia	137	2.3	109	28	20.4
Korea	135	2.3	122	13	9.6
France	113	1.9	95	18	15.9

**Figure 3:**
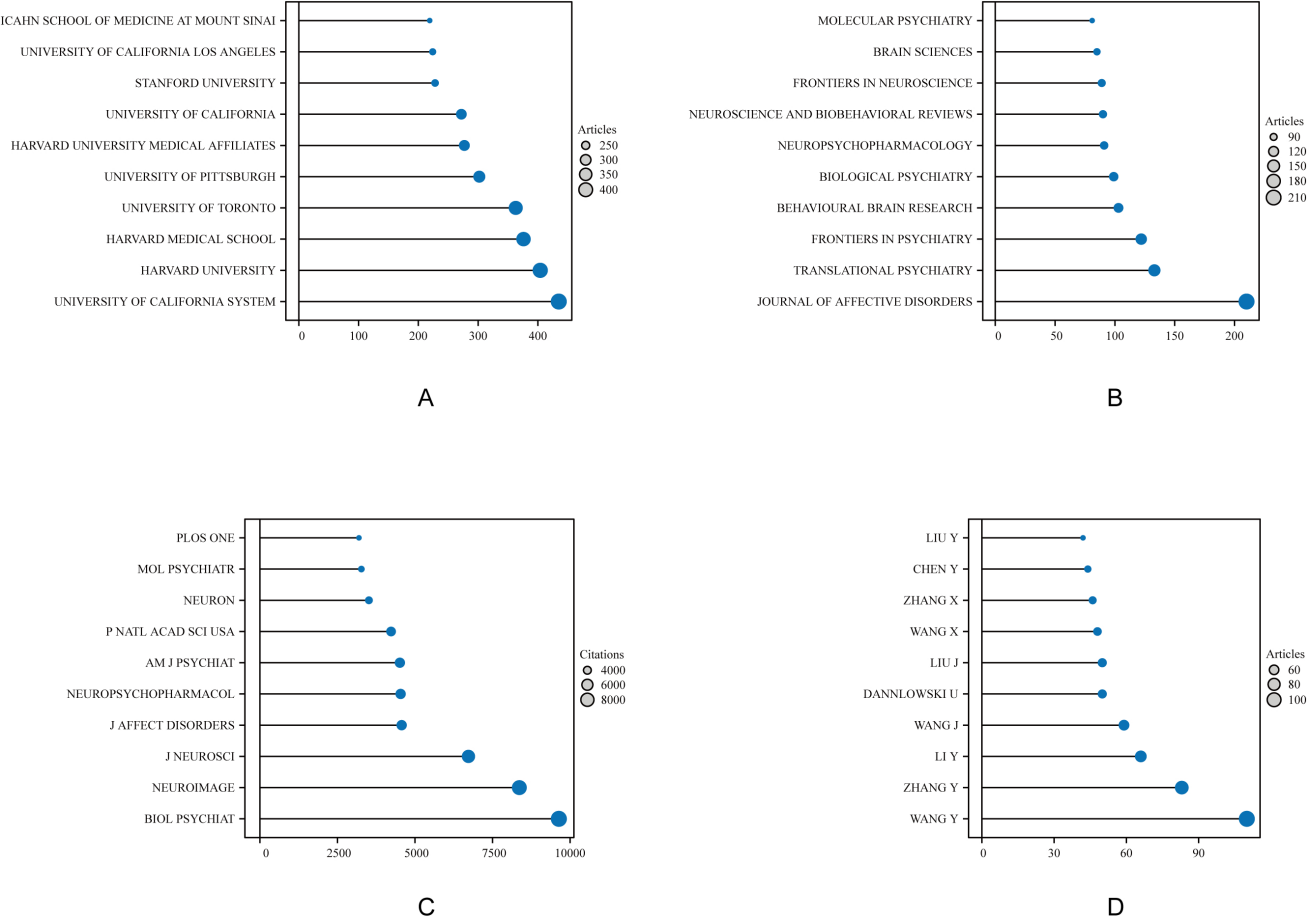
Top 10 institutions, journals, and authors in amygdala and depression research from 2015 to 2024. Each colored circle represents a distinct institution, journal, or author, with larger circles indicating higher counts. **(A)** Displays the top-ranking institutions by publication volume, reflecting their academic contribution and influence in this field. **(B)** Shows the journals with the highest number of publications, highlighting the core journals with strong academic impact. **(C)** Illustrates the top journals by total citation count, indicating their influence in knowledge dissemination and research recognition. **(D)** Depicts the top 10 most prolific authors, emphasizing their output and role in shaping this research domain.

In terms of institutional output, the top three institutions were the University of California System (N = 435), Harvard University (N = 403), and Harvard Medical School (N = 376), as shown in **Figure 3A**. A total of 1,037 journals published literature in this field, with the top 10 journals contributing 1,103 papers. The Journal of Affective Disorders published the most articles (N = 210), followed by Translational Psychiatry (N = 133), Frontiers in Psychiatry (N = 122), and Behavioral Brain Research (N = 103) (**Figure 3B**). The most cited journal was Biological Psychiatry (9,638 citations), followed by NeuroImage (8,364) and the Journal of Neuroscience (6,724) (**Figure 3C**). A total of 27,564 authors contributed to this research domain. The top five most productive authors were Wang Y (N = 110), Zhang Y (N = 83), Li Y (N = 66), Wang J (N = 59), and Dannlowski U (N = 50), collectively contributing 368 publications, accounting for 6.13% of the total output (**Figure 3D**).

### 3.3 Country and regional collaboration analysis

**Figure 4:**
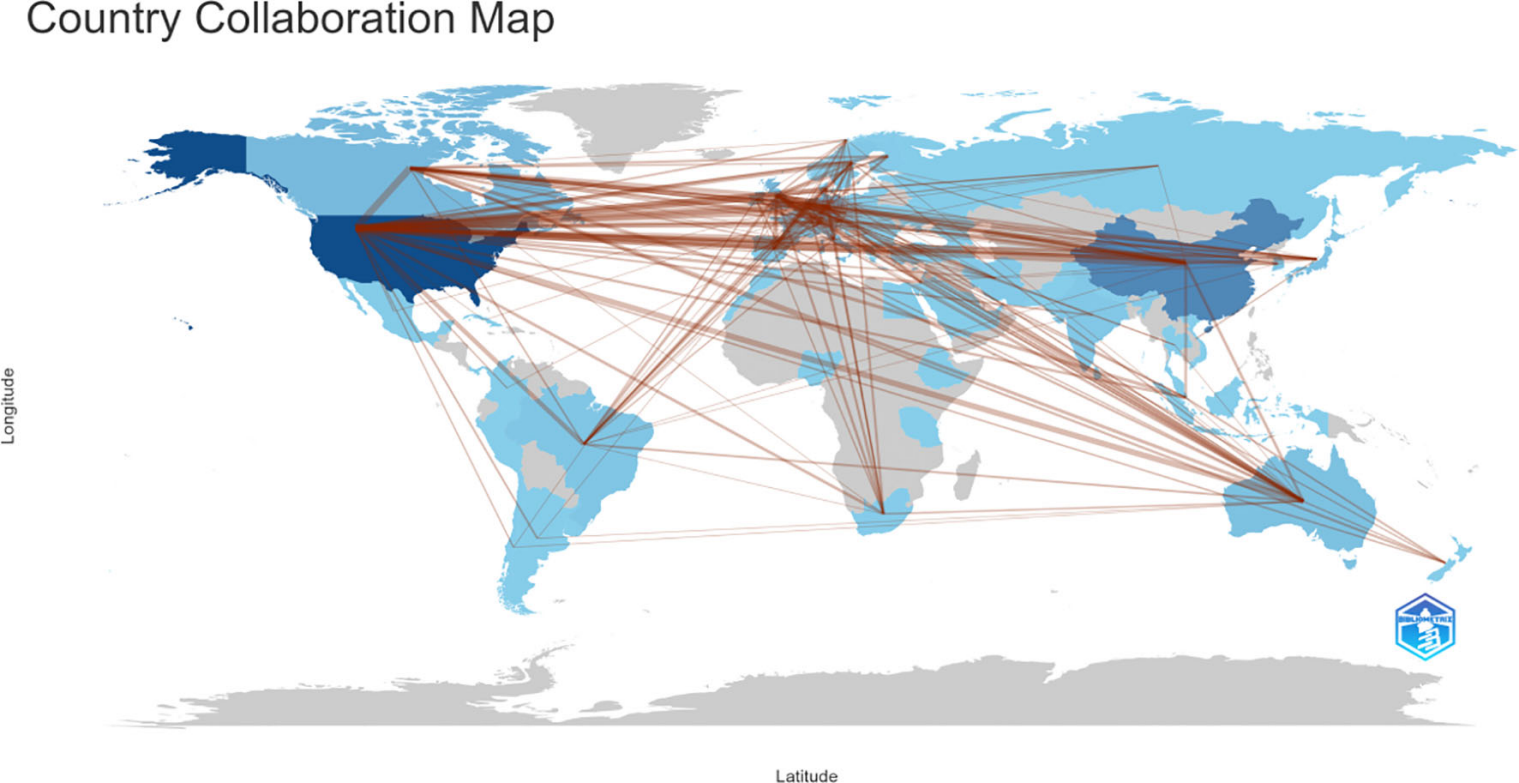
Country and regional collaboration in amygdala and depression research (2015–2024). The color segmentation includes blue (with publications) and grey (without publications). The thickness of the red lines indicates the number of co-published papers. The color intensity corresponds to the number of publications).

**Figure 4** presents the global collaboration network in amygdala–depression research. The figure uses a geographical visualization to illustrate inter-country scientific cooperation, where the density and distribution of connecting lines reflect collaboration intensity. As shown in **Figure 4**; **Table 2**, the United States occupies a central position in the international collaboration network, maintaining strong research ties with multiple countries. The most frequent collaboration occurred between the United States and China (n = 113), followed by the United Kingdom (n = 86), Germany (n = 81), and Canada (n = 78). The United States also maintained active partnerships with Australia (n = 51) and the Netherlands (n = 44). Within Europe, Germany demonstrated notable regional collaboration, particularly with Switzerland (n = 49), the United Kingdom (n = 48), and the Netherlands (n = 39). Overall, the international collaboration network was primarily concentrated in North America, Western Europe, East Asia, and Oceania, forming a multilateral structure centered on the United States and radiating toward Germany, China, the United Kingdom, and other key countries. Frequent collaborations between China and the United States, and between the United States and European countries, highlighted a high degree of international integration in this research domain, facilitating global knowledge exchange and advancing the study of amygdala–depression relationships.

**Table 2:** Top 10 collaboration pairs among countries in amygdala and depression research from 2015 to 2024.

From	To	Frequency
USA	China	113
USA	United Kingdom	86
USA	Germany	81
USA	Canada	78
USA	Australia	51
Germany	Switzerland	49
Germany	United Kingdom	48
USA	Netherlands	44
Germany	Netherlands	39
USA	Sweden	31

### 3.4 Global literature co-citation analysis

Global citations are a key bibliometric indicator, reflecting the total number of times a document has been cited across databases. They serve as a measure of a publication’s overall influence within the academic literature. In amygdala–depression research, the most globally cited publication was a 2017 review by Salim S., titled “Oxidative Stress and the Central Nervous System,” published in *Journal of Pharmacology and Experimental Therapeutics*, with 961 citations. This article emphasized the critical role of oxidative stress in central nervous system dysfunction. The second most cited work was by Foster J.A., also from 2017, titled “Stress and the Gut–Brain Axis: Regulation by the Microbiome,” published in *Neurobiology of Stress*, with 832 citations. It highlighted the role of gut microbiota in modulating stress responses through the gut–brain axis. Ranked third was a 2020 review by Van den Bergh B.R.H., titled “Prenatal Developmental Origins of Behavior and Mental Health: The Influence of Maternal Stress in Pregnancy,” published in *Neuroscience & Biobehavioral Reviews*, with 717 citations. This work systematically explored the long-term effects of prenatal stress on neurodevelopment and mental health outcomes (**Table 3**).

**Table 3:** Top 10 most globally cited publications in amygdala and depression research (2015–2024).

Number	First author	Article name	Journal name	Year	Global citations
1	Salim S.	Oxidative Stress and the Central Nervous System.	J Pharmacol Exp Ther	2017	961
2	Foster JA	Stress & the gut-brain axis: Regulation by the microbiome.	Neurobiol Stress.	2017	832
3	Van den Bergh BRH	Prenatal developmental origins of behavior and mental health: The influence of maternal stress in pregnancy.	Neurosci Biobehav Rev	2020	717
4	Jurek B	The Oxytocin Receptor: From Intracellular Signaling to Behavior.	Physiol Rev.	2018	658
5	Hiser J	The Multifaceted Role of the Ventromedial Prefrontal Cortex in Emotion, Decision Making, Social Cognition, and Psychopathology.	Biol Psychiatry.	2018	607
6	Grace AA	Dysregulation of the dopamine system in the pathophysiology of schizophrenia and depression.	Nat Rev Neurosci.	2016	598
7	Pinto-Sanchez MI	Probiotic Bifidobacterium longum NCC3001 Reduces Depression Scores and Alters Brain Activity: A Pilot Study in Patients With Irritable Bowel Syndrome.	Gastroenterology	2017	564
8	Bubb EJ	The cingulum bundle: Anatomy, function, and dysfunction.	Neurosci Biobehav Rev	2018	525
9	Liu W	The Role of Neural Plasticity in Depression: From Hippocampus to Prefrontal Cortex.	Neural Plast	2017	516
10	Belujon P	Dopamine System Dysregulation in Major Depressive Disorders.	Int J Neuropsychopharmacol	2017	508

### 3.5 Keyword co-occurrence and research hotspots

**Figure 5:**
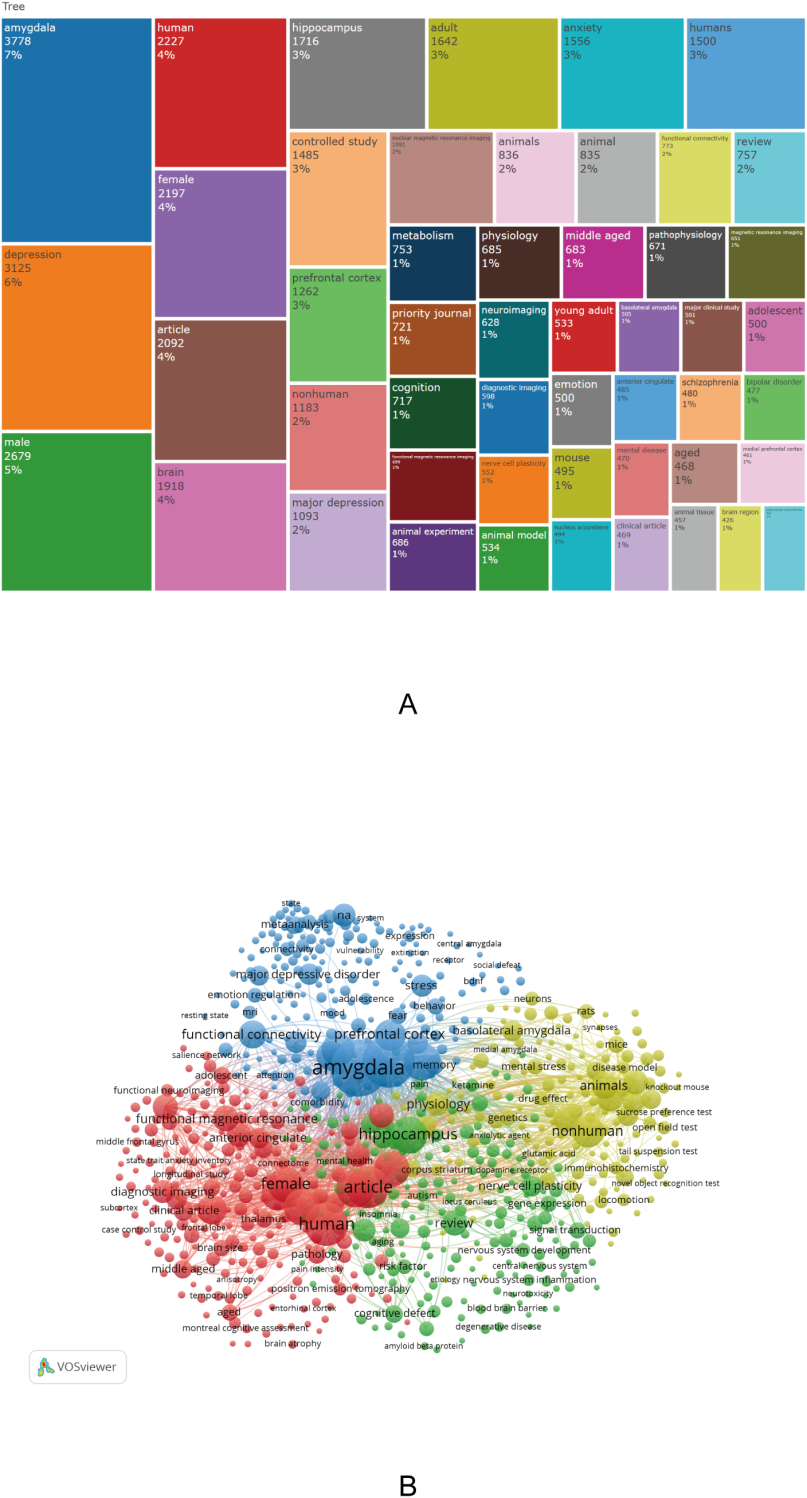
Keyword clustering analysis in depression–amygdala research. **(A)** Dendrogram of the top 50 keywords in the field. **(B)** Keyword co-occurrence cluster map (2015–2024) generated by *Bibliometrix*.

To visually demonstrate the distribution of research themes in the field of amygdala and depression, word cloud and pie charts were generated based on author keywords. Additionally, the “KeyWords Plus” field was extracted using *Bibliometrix*, yielding a total of 20,355 keywords. High-frequency terms were primarily related to brain structure, psychiatric disorders, population characteristics, and research methodologies. The most frequently occurring keywords were “amygdala” (3,778 occurrences, 7%) and “depression” (3,125 occurrences, 6%), underscoring their central role in this research area. Other frequently occurring terms included “male” (2,679), “female” (2,197), “brain” (1,918), “hippocampus” (1,716), “anxiety” (1,556), “controlled study” (1,485), and “prefrontal cortex” (1,262), reflecting a strong research focus on sex differences, emotional disorders, and the relationship between brain structure and function (**Figure 5A**).

In addition, a comprehensive keyword co-occurrence analysis was conducted using *VOSviewer*, with the parameters set to “All keywords” and “Full counting.” A total of 750 keywords with a minimum occurrence of 40 were identified and grouped into four thematic clusters, visualized in red, green, blue, and yellow, reflecting the multidimensional thematic structure of the field (**Figure 5B**). Cluster 1 (Red) focuses on neuroimaging features, abnormal brain function, and cognitive impairment; Cluster 2 (Green) emphasizes neurotransmitter regulation, gene expression, and pharmacological mechanisms, reflecting the biological underpinnings of depression; Cluster 3 (Blue) highlights emotional regulation and developmental risk factors, particularly the impact of early-life adversity on emotion processing and amygdala function; and Cluster 4 (Yellow) comprises animal model–based experimental research, focusing on neural circuitry, endocrine regulation, and behavioral mechanisms. Collectively, these results indicate that research on amygdala–depression spans multiple domains, including neuroimaging, biological mechanisms, psychological development, and experimental neuroscience.

## 4 Discussion

In recent years, research on the amygdala and depression has shown a marked increase and conceptual shift. The bibliometric results of this study reveal that global publications have risen sharply since 2020, with research focus gradually moving from single region functional analyses toward multilevel explorations of neural networks and systemic mechanisms. This trend reflects not only advances in neuroimaging and data analytics but also a growing consensus within the scientific community that the pathophysiology of depression has shifted from a “localized abnormality” model to a “systems integration” framework. Close collaboration among research institutions in the United States, China, and Europe has further accelerated this transition, propelling the field from fragmented studies toward a globally connected and mechanistically integrated stage of development.

### 4.1 Global landscape and research evolution: from regional abnormalities to systemic integration

The continuous expansion of research on depression and the amygdala reflects an increasingly sophisticated understanding of the core emotional circuitry underlying mood disorders. Early investigations primarily focused on structural alterations or activation differences within the amygdala, whereas more recent high-impact studies have revealed broader system-level mechanisms. Salim (2017) identified oxidative stress as a key contributor to central nervous system dysfunction (42); Foster (2017) and Pinto-Sanchez (2017) extended this concept to the gut–brain axis, emphasizing that microbiota can influence amygdala activity through immune and metabolic pathways, thereby modulating emotional regulation (43, 44). Van den Bergh (2020) demonstrated that prenatal stress disrupts the development of amygdala–prefrontal circuits, providing a neurodevelopmental basis for early preventive interventions (45).

In addition, several pivotal studies have expanded this mechanistic framework by linking the amygdala to neurotransmitter and structural pathways. Jurek (2018) and Hiser (2018) revealed that oxytocin and its receptor modulate connectivity between the amygdala and ventromedial prefrontal cortex (vmPFC), offering potential therapeutic targets (46, 47). Grace (2016) and Belujon (2017) reported that anhedonia in depression may stem from dysregulation of dopaminergic input from the amygdala and ventral hippocampus (48, 49). Structurally, Bubb et al. (2018) highlighted the cingulum bundle as a crucial tract linking the amygdala to the frontal lobe, suggesting that its disruption may underlie impaired emotion–cognition integration (50). Collectively, these findings converge on the amygdala as a multimodal hub integrating oxidative, immune, endocrine, and neurocircuit mechanisms—laying the groundwork for emerging systems-level frameworks of depression.

Consequently, the neurobiological model of depression has evolved from a single-region abnormality paradigm to a cross-system dysregulation framework, in which oxidative stress, inflammation, hormones, and neurotransmitters converge on the amygdala to jointly modulate emotion and cognition. This mechanistic transition parallels the bibliometric trends observed in this study, with a marked increase in interdisciplinary collaborations and multimodal imaging projects since 2020. These initiatives emphasize the interplay among functional connectivity, inflammatory signaling, and behavioral phenotypes, reflecting a collective shift in the field—from identifying isolated abnormalities to elucidating integrated mechanisms and constructing multilevel explanatory models.

### 4.2 Thematic clusters and scientific implications: integrating multi-level mechanisms

Keyword clustering analysis revealed four major thematic clusters within the depression–amygdala research field. These clusters are not independent but together form a continuum that bridges neural network dynamics, molecular regulation, developmental processes, and behavioral manifestations.

Cluster 1 focuses on resting-state functional connectivity and emotion-regulation networks, highlighting dysregulation within the prefrontal–amygdala circuitry. Numerous studies have demonstrated reduced amygdala–prefrontal and amygdala–limbic connectivity in patients with depression, indicating impaired top-down emotional control (47, 51–54). Such “prefrontal inhibition failure” corresponds closely to the negative emotional bias characteristic of depression and represents one of the most consistent neuroimaging signatures across studies. Cluster 2 encompasses molecular and neuroregulatory mechanisms, emphasizing the coupling between emotional circuits and neurotransmitter systems. Abnormal amygdala–ventral hippocampal connectivity has been linked to dopaminergic imbalance, providing a neural basis for anhedonia (46, 49). Oxytocin signaling enhances amygdala–prefrontal coupling, suggesting a potential neurobiological target for emotion regulation (46, 47). Meanwhile, chronic stress–induced microglial activation within the amygdala supports an inflammatory mechanism by which impaired synaptic plasticity contributes to mood dysregulation (55, 56). Cluster 3 addresses early-life stress and developmental emotion regulation, underscoring how environmental adversity shapes neural development. Prenatal and childhood stress have been shown to alter amygdala maturation and functional connectivity, establishing early emotional vulnerability (45, 57–59). Maternal depression, prenatal stress, and early abuse are all associated with disrupted amygdala–prefrontal connectivity in offspring, providing a neural basis for the intergenerational transmission of depression risk (45, 57, 60). Cluster 4 concerns animal models and systemic regulation, emphasizing the integration of physiological systems and neural circuitry. Animal experiments have demonstrated that oxidative stress, gut microbiota, glutamatergic transmission, and oxytocin signaling can modulate amygdala activity, providing direct evidence for neuro-immune-metabolic integration in depression pathophysiology (61–63). Taken together, these four thematic clusters delineate a hierarchical framework spanning molecular, neural, and behavioral levels. They underscore the amygdala’s pivotal role as an integrative hub within multidimensional regulatory networks and provide empirical foundations for the unified theoretical model proposed in the next section.

### 4.3 Theoretical integration: the amygdala as a cross-level emotional hub

Integrating bibliometric findings with mechanistic evidence, this study proposes a unified framework positioning the amygdala as a key intermediary node linking molecular biology, neural circuitry, and emotional behavior. Abnormalities in inflammation, stress hormones, and neurotransmission may alter the balance among the frontoparietal network (FPN), default mode network (DMN), and salience network (SN) through amygdala-centered modulation, leading to impaired emotional regulation and core depressive symptoms such as anhedonia.

Within this framework, the neuropathological features of depression can be understood as an imbalance between heightened emotional reactivity and weakened cognitive regulation. The amygdala and related limbic structures exhibit excessive sensitivity to negative emotional stimuli, persistently transmitting amplified negative signals to cortical regions. Meanwhile, the prefrontal cortex—particularly the dorsolateral prefrontal cortex (DLPFC) and ventromedial prefrontal cortex (vmPFC)—shows diminished regulatory and inhibitory control, failing to constrain amygdala hyperactivation. This “enhanced emotion generation–limited regulation” pattern of circuit-level dysfunction has been validated by multiple neuroimaging studies (15, 64, 65). Functional MRI research has demonstrated heightened amygdala responses to negative stimuli in patients with depression (15, 64), along with reduced prefrontal activation during cognitive reappraisal or inhibitory tasks (64). Meta-analyses and resting-state studies further reveal decreased amygdala–prefrontal connectivity, reflecting impaired emotional control (66, 67). Comprehensive reviews support this framework, suggesting that hyperactive emotion-processing networks maintain persistent negative affect, while inefficient prefrontal regulation amplifies sustained negative thinking and cognitive bias (65, 68).

Therefore, amygdala-based functional dysconnectivity represents more than regional hyperactivation—it reflects a breakdown in the coordination between emotional generation and cognitive control systems. This circuit imbalance provides a coherent neurobiological explanation for the heterogeneity and symptom persistence observed in depression. In summary, the amygdala has evolved from a passive marker of emotional response to an active hub of emotional regulation and therapeutic intervention. This integrative model offers new insights into the multidimensional neuropathology of depression and establishes a theoretical foundation for precision diagnosis and circuit-targeted interventions in affective disorders.

### 4.4 Clinical and translational implications: from neural biomarkers to therapeutic targets

Current evidence indicates that amygdala functional connectivity has three advantages as a candidate biomarker for depression: it is quantifiable, interpretable, and amenable to intervention. First, in diagnosis and patient stratification, individual differences in resting-state amygdala connectivity can help identify age- and sex-specific neural signatures across different disease stages (52, 53). When combined with peripheral inflammatory markers or neurotransmitter profiles, these connectivity alterations may serve as cross-modal biomarkers, bridging brain imaging and molecular indices to refine diagnostic precision.

Second, in prognosis and treatment prediction, baseline amygdala–insula connectivity strength has been shown to predict subsequent symptom remission and treatment responsiveness (54). This suggests that circuit-level metrics could be used as dynamic indicators for tracking therapeutic outcomes and guiding personalized interventions.

Third, in intervention and neuroplasticity modulation, noninvasive neuromodulation techniques—such as repetitive transcranial magnetic stimulation (rTMS), transcranial direct current stimulation (tDCS), theta-burst stimulation (TBS), and real-time fMRI neurofeedback—can effectively restore the functional integrity of the prefrontal–amygdala circuitry. Preclinical findings further support this translational potential: animal studies have demonstrated that oxytocin and glutamatergic pathways modulate amygdala excitability, providing mechanistic targets for circuit-specific therapies (46, 47, 61, 62).

In parallel, advances in artificial intelligence (AI) and the Internet of Medical Things (IoMT) are paving the way for the digitization of amygdala-based biomarkers (34, 39). Functional connectivity features may soon be transformed into computational indicators for multimodal prediction, remote monitoring, and relapse prevention. Establishing standardized protocols for imaging acquisition, preprocessing, and data sharing across centers will be essential for ensuring reproducibility and scalability. Collectively, these developments highlight the translational promise of amygdala-centered neural markers in achieving precision prevention, personalized intervention, and long-term management of depressive disorders.

### 4.5 Limitations and future directions

Several limitations should be acknowledged in this study. First, the use of only the Web of Science Core Collection (WoS) and Scopus databases may have introduced coverage bias. Second, the restriction to English-language publications and a fixed search time window may have excluded recent or non-English studies. Third, differences in cross-database deduplication procedures and parameter settings may affect the precision of clustering results. Moreover, this study did not perform country-level thematic comparisons to avoid attribution bias caused by multi-institutional and multi-country authorship. Finally, variations in citation definitions and threshold criteria across databases prevented the listing of highly cited papers within each cluster.

Despite these limitations, this study integrated global bibliometric evidence with mechanistic research findings to reveal the systematic evolution and knowledge structure of depression–amygdala research. Future research should focus on three key directions: (i) longitudinal and multimodal validation of the amygdala’s causal role as a neural regulatory hub; (ii) construction of multicenter imaging–molecular–behavioral databases to identify replicable and generalizable neural biomarker systems; and (iii) development of individualized intervention models based on amygdala functional connectivity, achieving a closed loop from mechanistic understanding to precision treatment.

Overall, abnormalities in amygdala functional connectivity reflect a system-level disequilibrium spanning from molecular to network levels in depression. The amygdala has thus evolved from a passive structure of emotional reactivity to an active integrative hub in affective regulation. The findings of this study not only map the intellectual evolution of the field but also provide a theoretical foundation for future research on multidimensional mechanisms and circuit-guided interventions in affective disorders.

## 5 Conclusion

Using data from the Web of Science Core Collection and Scopus, this bibliometric study mapped the 2015–2024 landscape of amygdala-related depression research. Publication output rose markedly after 2020, and the field has shifted from single-region accounts to systems-level frameworks. Keyword and clustering results converge on four themes: emotion-regulation networks, molecular and neural regulation, developmental risk factors, and animal models. Across these themes, amygdala functional connectivity emerges as a promising candidate neuromarker for stratification and treatment prediction. Despite limitations (database scope, language/time window, cross-database harmonization), the overall trajectory points to mechanistic precision and system integration. Future work should adopt harmonized, multicenter, multimodal designs with age/sex stratification, and integrate peripheral biomarkers and AI-enabled analytics to translate candidate markers into scalable clinical readouts.

## Data Availability

The original contributions presented in the study are included in the article/**Supplementary Material**, further inquiries can be directed to the corresponding author/s. The data supporting this study are available from the Web of Science and Scopus databases.

## References

[1] Reed GM First MB Kogan CS Hyman SE Gureje O Gaebel W . Innovations and changes in the ICD-11 classification of mental, behavioural and neurodevelopmental disorders. World Psychiatry. (2019) 18:3–19. doi: 10.1002/wps.20611, PMID: 30600616 PMC6313247

[2] Global, regional, and national burden of 12 mental disorders in 204 countries and territories, 1990-2019: a systematic analysis for the Global Burden of Disease Study 2019. Lancet Psychiatry. (2022) 9:137–50. doi: 10.1016/S2215-0366(21)00395-3, PMID: 35026139 PMC8776563

[3] Global prevalence and burden of depressive and anxiety disorders in 204 countries and territories in 2020 due to the COVID-19 pandemic. Lancet (London England). (2021) 398:1700–12. doi: 10.1016/S0140-6736(21)02143-7, PMID: 34634250 PMC8500697

[4] Li S Guo B Yang Q Yin J Jiang Y Tian L . Factors associated with depression in residents in the post-epidemic era. QJM. (2022) 115:605–9. doi: 10.1093/qjmed/hcac181, PMID: 35900167 PMC9384610

[5] Monroe SM Harkness KL . Major depression and its recurrences: life course matters. Annu Rev Clin Psychol. (2022) 18:329–57. doi: 10.1146/annurev-clinpsy-072220-021440, PMID: 35216520

[6] Shorey S Ng ED Wong CHJ . Global prevalence of depression and elevated depressive symptoms among adolescents: A systematic review and meta-analysis. Br J Clin Psychol. (2022) 61:287–305. doi: 10.1111/bjc.12333, PMID: 34569066

[7] Malhi GS Mann JJ . Depression. Lancet (London England). (2018) 392:2299–312. doi: 10.1016/S0140-6736(18)31948-2, PMID: 30396512

[8] Wang Y Jiang G Wang L Chen M Yang K Wen K . Association of the depressive scores, depressive symptoms, and conversion patterns of depressive symptoms with the risk of new-onset chronic diseases and multimorbidity in the middle-aged and elderly Chinese population. EClinicalMedicine. (2022) 52:101603. doi: 10.1016/j.eclinm.2022.101603, PMID: 35958523 PMC9358433

[9] Dibeklioglu H Hammal Z Cohn JF . Dynamic multimodal measurement of depression severity using deep autoencoding. IEEE J Biomed Health Inf. (2018) 22:525–36. doi: 10.1109/JBHI.2017.2676878, PMID: 28278485 PMC5581737

[10] Phelps EA LeDoux JE . Contributions of the amygdala to emotion processing: from animal models to human behavior. Neuron. (2005) 48:175–87. doi: 10.1016/j.neuron.2005.09.025, PMID: 16242399

[11] Šimić G Tkalčić M Vukić V Mulc D Španić E Šagud M . Understanding emotions: origins and roles of the amygdala. Biomolecules. (2021) 11:823. doi: 10.3390/biom11060823, PMID: 34072960 PMC8228195

[12] Ghaemi Kerahrodi J Michal M . The fear-defense system, emotions, and oxidative stress. Redox Biol. (2020) 37:101588. doi: 10.1016/j.redox.2020.101588, PMID: 32739155 PMC7767737

[13] Paz R Pare D . Physiological basis for emotional modulation of memory circuits by the amygdala. Curr Opin Neurobiol. (2013) 23:381–6. doi: 10.1016/j.conb.2013.01.008, PMID: 23394774 PMC3652906

[14] Zhong M Wang X Xiao J Yi J Zhu X Liao J . Amygdala hyperactivation and prefrontal hypoactivation in subjects with cognitive vulnerability to depression. Biol Psychol. (2011) 88:233–42. doi: 10.1016/j.biopsycho.2011.08.007, PMID: 21878364

[15] Sheline YI Barch DM Donnelly JM Ollinger JM Snyder AZ Mintun MA . Increased amygdala response to masked emotional faces in depressed subjects resolves with antidepressant treatment: an fMRI study. Biol Psychiatry. (2001) 50:651–8. doi: 10.1016/S0006-3223(01)01263-X, PMID: 11704071

[16] Fu CH Williams SC Cleare AJ Scott J Mitterschiffthaler MT Walsh ND . Neural responses to sad facial expressions in major depression following cognitive behavioral therapy. Biol Psychiatry. (2008) 64:505–12. doi: 10.1016/j.biopsych.2008.04.033, PMID: 18550030

[17] Fu CH Williams SC Cleare AJ Brammer MJ Walsh ND Kim J . Attenuation of the neural response to sad faces in major depression by antidepressant treatment: a prospective, event-related functional magnetic resonance imaging study. Arch Gen Psychiatry. (2004) 61:877–89. doi: 10.1001/archpsyc.61.9.877, PMID: 15351766

[18] Suslow T Konrad C Kugel H Rumstadt D Zwitserlood P Schöning S . Automatic mood-congruent amygdala responses to masked facial expressions in major depression. Biol Psychiatry. (2010) 67:155–60. doi: 10.1016/j.biopsych.2009.07.023, PMID: 19748075

[19] Victor TA Furey ML Fromm SJ Ohman A Drevets WC . Relationship between amygdala responses to masked faces and mood state and treatment in major depressive disorder. Arch Gen Psychiatry. (2010) 67:1128–38. doi: 10.1001/archgenpsychiatry.2010.144, PMID: 21041614 PMC3253452

[20] Abler B Erk S Herwig U Walter H . Anticipation of aversive stimuli activates extended amygdala in unipolar depression. J Psychiatr Res. (2007) 41:511–22. doi: 10.1016/j.jpsychires.2006.07.020, PMID: 17010993

[21] Kessler H Taubner S Buchheim A Münte TF Stasch M Kächele H . Individualized and clinically derived stimuli activate limbic structures in depression: an fMRI study. PloS One. (2011) 6:e15712. doi: 10.1371/journal.pone.0015712, PMID: 21283580 PMC3026801

[22] Siegle GJ Thompson W Carter CS Steinhauer SR Thase ME . Increased amygdala and decreased dorsolateral prefrontal BOLD responses in unipolar depression: related and independent features. Biol Psychiatry. (2007) 61:198–209. doi: 10.1016/j.biopsych.2006.05.048, PMID: 17027931

[23] Carballedo A Scheuerecker J Meisenzahl E Schoepf V Bokde A Möller HJ . Functional connectivity of emotional processing in depression. J Affect Disord. (2011) 134:272–9. doi: 10.1016/j.jad.2011.06.021, PMID: 21757239

[24] Chen CH Suckling J Ooi C Fu CH Williams SC Walsh ND . Functional coupling of the amygdala in depressed patients treated with antidepressant medication. Neuropsychopharmacology. (2008) 33:1909–18. doi: 10.1038/sj.npp.1301593, PMID: 17987064

[25] Dannlowski U Ohrmann P Konrad C Domschke K Bauer J Kugel H . Reduced amygdala-prefrontal coupling in major depression: association with MAOA genotype and illness severity. Int J Neuropsychopharmacol. (2009) 12:11–22. doi: 10.1017/S1461145708008973, PMID: 18544183

[26] Hakamata Y Mizukami S Izawa S Hori H Matsui M Moriguchi Y . Contextual memory bias in emotional events: Neurobiological correlates and depression risk. Psychoneuroendocrinology. (2025) 171:107218. doi: 10.1016/j.psyneuen.2024.107218, PMID: 39531919

[27] Tottenham N Weissman MM Wang Z Warner V Gameroff MJ Semanek DP . Depression risk is associated with weakened synchrony between the amygdala and experienced emotion. Biol Psychiatry Cogn Neurosci Neuroimaging. (2021) 6:343–51. doi: 10.1016/j.bpsc.2020.10.011, PMID: 33487578 PMC7946704

[28] Hakamata Y Mizukami S Izawa S Okamura H Mihara K Marusak H . Implicit and explicit emotional memory recall in anxiety and depression: Role of basolateral amygdala and cortisol-norepinephrine interaction. Psychoneuroendocrinology. (2022) 136:105598. doi: 10.1016/j.psyneuen.2021.105598, PMID: 34894424

[29] Tani Y Koyama Y Doi S Sugihara G Machida M Amagasa S . Association between gratitude, the brain and cognitive function in older adults: Results from the NEIGE study. Arch Gerontology Geriatrics. (2022) 100:104645. doi: 10.1016/j.archger.2022.104645, PMID: 35123174

[30] Erdmann T Berwian IM Stephan KE Seifritz E Walter H Huys QJM . Amygdala reactivity, antidepressant discontinuation, and relapse. JAMA Psychiatry. (2024) 81:1081–9. doi: 10.1001/jamapsychiatry.2024.2136, PMID: 39259548 PMC11391364

[31] Jin L Wu L Zhang J Jia W Zhou H Jiang S . Quantitative analysis of literature on diagnostic biomarkers of Schizophrenia: revealing research hotspots and future prospects. BMC Psychiatry. (2025) 25:186. doi: 10.1186/s12888-025-06644-3, PMID: 40025442 PMC11872302

[32] He T Wang D Wu L Jin L . The role of glial cells in neuralgia: a bibliometric exploration. Front Neurol. (2025) 16:1496526. doi: 10.3389/fneur.2025.1496526, PMID: 39990265 PMC11842238

[33] Akhtar F Belal Bin Heyat M Sultana A Parveen S Muhammad Zeeshan H Merlin SF . Medical intelligence for anxiety research: Insights from genetics, hormones, implant science, and smart devices with future strategies. WIREs Data Min Knowledge Discov. (2024) 14:e1552. doi: 10.1002/widm.1552

[34] Heyat MBB Akhtar F Munir F Sultana A Muaad AY Gul I . Unravelling the complexities of depression with medical intelligence: exploring the interplay of genetics, hormones, and brain function. Complex Intelligent Syst. (2024) 10:5883–915. doi: 10.1007/s40747-024-01346-x

[35] Falagas ME Pitsouni EI Malietzis GA Pappas G . Comparison of PubMed, Scopus, Web of Science, and Google Scholar: strengths and weaknesses. FASEB J. (2008) 22:338–42. doi: 10.1096/fj.07-9492LSF, PMID: 17884971

[36] Gusenbauer M . Beyond Google Scholar, Scopus, and Web of Science: An evaluation of the backward and forward citation coverage of 59 databases' citation indices. Res Synthesis Methods. (2024) 15:802–17. doi: 10.1002/jrsm.1729, PMID: 38877607

[37] Martín-Martín A Thelwall M Orduna-Malea E Delgado López-Cózar E . Google Scholar, Microsoft Academic, Scopus, Dimensions, Web of Science, and OpenCitations' COCI: a multidisciplinary comparison of coverage via citations. Scientometrics. (2021) 126:871–906. doi: 10.1007/s11192-020-03690-4, PMID: 32981987 PMC7505221

[38] Heyat MBB Akhtar F Khan MH Ullah N Gul I Khan H . Detection, treatment planning, and genetic predisposition of bruxism: A systematic mapping process and network visualization technique. CNS Neurological Disord Drug Targets. (2021) 20:755–75. doi: 10.2174/19963181MTExyMzM33, PMID: 33172381

[39] Zeeshan HM Heyat MBB Hayat MAB Parveen S Akhtar F Sayeed E . (2023). Worldwide research trends and hotspot on IOMT based on bibliometric analysis, in: 2023 20th International Computer Conference on Wavelet Active Media Technology and Information Processing (ICCWAMTIP), Chengdu, China: IEEE, 2023 15–17 Dec.

[40] Gustavsson EK Zhang D Reynolds RH Garcia-Ruiz S Ryten M . ggtranscript: an R package for the visualization and interpretation of transcript isoforms using ggplot2. Bioinf (Oxford England). (2022) 38:3844–6. doi: 10.1101/2022.03.28.486050, PMID: 35751589 PMC9344834

[41] Van Eck NJ Waltman L . Software survey: VOSviewer, a computer program for bibliometric mapping. Scientometrics. (2010) 84:523–38. doi: 10.1007/s11192-009-0146-3, PMID: 20585380 PMC2883932

[42] Salim S . Oxidative stress and the central nervous system. J Pharmacol Exp Ther. (2017) 360:201–5. doi: 10.1124/jpet.116.237503, PMID: 27754930 PMC5193071

[43] Foster JA Rinaman L Cryan JF . Stress & the gut-brain axis: Regulation by the microbiome. Neurobiol Stress. (2017) 7:124–36. doi: 10.1016/j.ynstr.2017.03.001, PMID: 29276734 PMC5736941

[44] Pinto-Sanchez MI Hall GB Ghajar K Nardelli A Bolino C Lau JT . Probiotic bifidobacterium longum NCC3001 reduces depression scores and alters brain activity: A pilot study in patients with irritable bowel syndrome. Gastroenterology. (2017) 153:448–59.e8. doi: 10.1053/j.gastro.2017.05.003, PMID: 28483500

[45] Van Den Bergh BRH Van Den Heuvel MI Lahti M Braeken M De Rooij SR Entringer S . Prenatal developmental origins of behavior and mental health: The influence of maternal stress in pregnancy. Neurosci Biobehav Rev. (2020) 117:26–64. doi: 10.1016/j.neubiorev.2017.07.003, PMID: 28757456

[46] Jurek B Neumann ID . The oxytocin receptor: from intracellular signaling to behavior. Physiol Rev. (2018) 98:1805–908. doi: 10.1152/physrev.00031.2017, PMID: 29897293

[47] Hiser J Koenigs M . The multifaceted role of the ventromedial prefrontal cortex in emotion, decision making, social cognition, and psychopathology. Biol Psychiatry. (2018) 83:638–47. doi: 10.1016/j.biopsych.2017.10.030, PMID: 29275839 PMC5862740

[48] Grace AA . Dysregulation of the dopamine system in the pathophysiology of schizophrenia and depression. Nat Rev Neurosci. (2016) 17:524–32. doi: 10.1038/nrn.2016.57, PMID: 27256556 PMC5166560

[49] Belujon P Grace AA . Dopamine system dysregulation in major depressive disorders. Int J Neuropsychopharmacol. (2017) 20:1036–46. doi: 10.1093/ijnp/pyx056, PMID: 29106542 PMC5716179

[50] Bubb EJ Metzler-Baddeley C Aggleton JP . The cingulum bundle: Anatomy, function, and dysfunction. Neurosci Biobehav Rev. (2018) 92:104–27. doi: 10.1016/j.neubiorev.2018.05.008, PMID: 29753752 PMC6090091

[51] Ramasubbu R Konduru N Cortese F Bray S Gaxiola-Valdez I Goodyear B . Reduced intrinsic connectivity of amygdala in adults with major depressive disorder. Front Psychiatry. (2014) 5:17. doi: 10.3389/fpsyt.2014.00017, PMID: 24600410 PMC3928548

[52] Guan L Liu R Wang C Fan Q Zhou J Wang Y . Abnormal resting-state functional connectivity in subregions of amygdala in adults and adolescents with major depressive disorder. BMC Psychiatry. (2024) 24:540. doi: 10.1186/s12888-024-05977-9, PMID: 39085839 PMC11293025

[53] Tang S Lu L Zhang L Hu X Bu X Li H . Abnormal amygdala resting-state functional connectivity in adults and adolescents with major depressive disorder: A comparative meta-analysis. EBioMedicine. (2018) 36:436–45. doi: 10.1016/j.ebiom.2018.09.010, PMID: 30316866 PMC6197798

[54] Connolly CG Ho TC Blom EH LeWinn KZ Sacchet MD Tymofiyeva O . Resting-state functional connectivity of the amygdala and longitudinal changes in depression severity in adolescent depression. J Affect Disord. (2017) 207:86–94. doi: 10.1016/j.jad.2016.09.026, PMID: 27716542 PMC5149416

[55] Calcia MA Bonsall DR Bloomfield PS Selvaraj S Barichello T Howes OD . Stress and neuroinflammation: a systematic review of the effects of stress on microglia and the implications for mental illness. Psychopharmacology. (2016) 233:1637–50. doi: 10.1007/s00213-016-4218-9, PMID: 26847047 PMC4828495

[56] Zheng ZH Tu JL Li XH Hua Q Liu WZ Liu Y . Neuroinflammation induces anxiety- and depressive-like behavior by modulating neuronal plasticity in the basolateral amygdala. Brain Behavior Immun. (2021) 91:505–18. doi: 10.1016/j.bbi.2020.11.007, PMID: 33161163

[57] Uy JP Tan AP Broeckman B Gluckman PD Chong YS Chen H . Effects of maternal childhood trauma on child emotional health: maternal mental health and frontoamygdala pathways. J Child Psychol Psychiatry Allied Disciplines. (2023) 64:426–36. doi: 10.1111/jcpp.13721, PMID: 36331294

[58] Teicher MH Samson JA . Annual Research Review: Enduring neurobiological effects of childhood abuse and neglect. J Child Psychol Psychiatry Allied Disciplines. (2016) 57:241–66. doi: 10.1111/jcpp.12507, PMID: 26831814 PMC4760853

[59] Nogovitsyn N Addington J Souza R Placsko TJ Stowkowy J Wang J . Childhood trauma and amygdala nuclei volumes in youth at risk for mental illness. psychol Med. (2022) 52:1192–9. doi: 10.1017/S0033291720003177, PMID: 32940197

[60] Qiu A Anh TT Li Y Chen H Rifkin-Graboi A Broekman BF . Prenatal maternal depression alters amygdala functional connectivity in 6-month-old infants. Trans Psychiatry. (2015) 5:e508. doi: 10.1038/tp.2015.3, PMID: 25689569 PMC4445753

[61] Althammer F Roy RK Kirchner MK Lira EC Schimmer S Charlet A . Impaired oxytocin signalling in the central amygdala in rats with chronic heart failure. J Physiol. (2024) 602:6259–80. doi: 10.1113/JP286297, PMID: 39530490 PMC11576253

[62] Fazmiya MJA Sultana A Rahman K Heyat MBB Sumbul, Akhtar F . Current insights on bioactive molecules, antioxidant, anti-inflammatory, and other pharmacological activities of cinnamomum camphora linn. Oxid Med Cell Longevity. (2022) 2022:9354555. doi: 10.1155/2022/9354555, PMID: 36246399 PMC9568346

[63] He S Huang X Zheng J Zhang Y Ruan X . An NTS-CeA projection modulates depression-like behaviors in a mouse model of chronic pain. Neurobiol Dis. (2022) 174:105893. doi: 10.1016/j.nbd.2022.105893, PMID: 36229006

[64] Johnstone T Van Reekum CM Urry HL Kalin NH Davidson RJ . Failure to regulate: counterproductive recruitment of top-down prefrontal-subcortical circuitry in major depression. J Neurosci. (2007) 27:8877–84. doi: 10.1523/JNEUROSCI.2063-07.2007, PMID: 17699669 PMC6672169

[65] Disner SG Beevers CG Haigh EA Beck AT . Neural mechanisms of the cognitive model of depression. Nat Rev Neurosci. (2011) 12:467–77. doi: 10.1038/nrn3027, PMID: 21731066

[66] Etkin A Wager TD . Functional neuroimaging of anxiety: a meta-analysis of emotional processing in PTSD, social anxiety disorder, and specific phobia. Am J Psychiatry. (2007) 164:1476–88. doi: 10.1176/appi.ajp.2007.07030504, PMID: 17898336 PMC3318959

[67] Zeng LL Shen H Liu L Wang L Li B Fang P . Identifying major depression using whole-brain functional connectivity: a multivariate pattern analysis. Brain: J Neurol. (2012) 135:1498–507. doi: 10.1093/brain/aws059, PMID: 22418737

[68] Ressler KJ Mayberg HS . Targeting abnormal neural circuits in mood and anxiety disorders: from the laboratory to the clinic. Nat Neurosci. (2007) 10:1116–24. doi: 10.1038/nn1944, PMID: 17726478 PMC2444035

